# How does technology challenge teacher education?

**DOI:** 10.1186/s41239-022-00375-1

**Published:** 2022-12-27

**Authors:** Lina Kaminskienė, Sanna Järvelä, Erno Lehtinen

**Affiliations:** 1 Education Academy, Vytautas Magnus University, Kaunas, Lithuania; 2 Faculty of Education and Psychology, University of Oulu, Oulu, Finland; 3 Department of Teacher Education, University of Turku, Turku, Finland

**Keywords:** Teachers’ digital competences, Teachers’ initial and continuous training, Technology integration, Digital innovations in education

## Abstract

The paper presents an overview of challenges and demands related to teachers’ digital skills and technology integration into educational content and processes. The paper raises a debate how technologies have created new skills gaps in pre-service and in-service teacher training and how that affected traditional forms of teacher education. Accordingly, it is discussed what interventions might be applicable to different contexts to address these challenges. It is argued that technologies should be viewed both as the field where new competences should be developed and at the same time as the method used in developing learning environments for teacher students.

## Introduction

In the last few decades, national authorities and multinational organisations have emphasised the importance of increasing the use of information and communication technologies (ICT) in schools and universities (Flecknoe, 2002; Roztocki et al., 2019; UNESCO ICT Competency Framework for Teachers, 2018). This poses a double challenge for teacher education: determining how new technologies can be used to improve the quality of learning experiences that student teachers receive during their university studies and identifying what kinds of new skills future teachers will need for teaching in technologically rich school environments. Several of the arguments in favour of greater use of ICT in schools are based on the belief that due to the general digitisation of the workforce, it is vital that students acquire good digital skills at an early age. However, it has also been argued that the use of ICT will be engaging for students and can thus result in better learning outcomes (Cheung & Slavin, 2013; Gloria, 2015). A number of large meta-analyses have shown that intervention studies utilising technology have positive effects on students’ motivation and learning (Fadda et al., 2022; Wouters et al., 2013).

However, when large-scale national and international evaluation studies have examined the relationship between the use of ICT and student achievement, the results have been mixed. Researchers have reported that re-analyses of large international evaluation studies, including the Trends in International Mathematics and Science Study (TIMMS) and Programme for International Student Assessment (PISA), indicate that there is either no relationship or a negative relationship between the frequency of ICT use in teaching and students’ achievements (Eickelmann et al., 2016; Papanastasiou et al., 2003).

The mixed results regarding the impact of ICT suggest that there are qualitative differences between the ways in which technology is implemented. In intervention studies, ICT applications have typically been used with careful planning, with extensive professional development for teachers conducting the experiment and with continuous support from researchers, whereas large-scale evaluation studies have focused on regular classrooms without such support. When technology is implemented in these latter situations, the pedagogical quality of the technology application is determined by the teachers’ knowledge and skills. However, there is a great deal of variation in the knowledge and competences of teachers when it comes to using technology in their classrooms (Valtonen et al., 2016). This highlights the importance of the in-service training of teachers while at the same time calling attention to pre-service teachers’ opportunities to acquire the competencies necessary to implement technology in their classrooms.

What does the development of technology mean for teacher education and how does it challenge traditional forms and content of the discipline? During the past few decades, a large number of policy documents and scientific studies have addressed this issue (Bakir, 2015). Different perspectives can be taken into account when discussing the influence of technology on teacher education. Several studies have examined the skills that will be necessary to apply technology to pedagogical practice in the future. This topic has been explored from several perspectives, such as teacher students’ technology literacy or their knowledge of technological pedagogical content (Mishra & Koehler, 2006). To ensure that future teachers possess adequate technical skills, standards and recommendations have been developed regarding the content of teacher education programmes. Rather than simply focusing on basic technological skills, the main emphasis has been on the knowledge and skills associated with the pedagogical use of technology (Erstad et al., 2021). Moreover, technology can provide many opportunities to develop novel methods to improve the quality of teacher education, such as the development of new methods for conducting research in the field of teacher education. In this special issue, these issues are addressed from a variety of theoretical and methodological perspectives.

## Technology integration and content in teacher education

Given the many ways in which technology can be used in education, pre-service teachers’ pedagogical and technical competences in using ICT in teaching have different dimensions. For instance, Tondeur et al. (2017) developed a test to measure pre-service teachers’ ICT abilities and applied it to a large sample of Belgian teacher candidates. According to the findings, there are two dimensions to ICT competences: (1) competencies for supporting students’ use of ICT in class and (2) competencies for using ICT to create instructional materials. Some studies have also reported barriers that hinder the organisation of adequate teacher education to develop these skills, such as faculty beliefs and skills (Bakir, 2015; Polly et al., 2010).

The experiences pre-service teachers acquire during their teacher studies have also been shown to influence how willing and skilled they are when it comes to integrating technology in their classrooms (Agyei & Voogt, 2011). Additionally, studies have shown that the opportunity for teacher students to observe advanced technology applications in real-world settings is important for their future professional development (Gromseth et al., 2010). Hence, it is not sufficient to take formal courses in ICT or educational technology without applying these skills in the classroom.

Finnish pre-service teacher education stresses not only ICT skills but also teachers’ competences, such as strategic learning skills and collaboration competences. Häkkinen et al. (2017) identified five profiles among Finnish first-year pre-service teachers (N = 872) using perceptions of the teachers’ strategic learning skills and collaboration dispositions and investigated what background variables explained membership to those profiles. The most robust factor explaining membership in the profiles was life satisfaction. For example, pre-service teachers in a profile group with high strategic learning skills and high collaboration dispositions showed the highest anticipated life satisfaction after 5 years. Their results demonstrate the need to develop both ICT skills and learning competences in pre-service teacher education.

## Use of technological innovations in organising teacher education

An analysis of the nature of experience and how practise can optimally enhance expertise has demonstrated the importance of deliberate practise (Ericsson et al., 1993), defined as an intensive practise that is purposefully focused on developing specific aspects of performance. To achieve this, it is necessary to have the opportunity to practise the most demanding aspects of performance with a large number of repetitions. Feedback from a tutor or coach also plays an important role in deliberate practise (Ericsson, 1993). Although deliberate practise has already been applied in some teacher education studies (e.g. Bronkhorst, 2011), its main aspect, repeated practise of challenging tasks and informed feedback, has been difficult to apply in traditional teacher education settings. Recent studies have shown that technology can contribute to the development of training methods that better reflect the main principles of deliberate practise.

There is a long tradition of using video technology in teacher education; such technology was first applied in a systematic manner in the 1970s (Nagro & Cornelius, 2013). Since then, a number of video-assisted instructional designs have been developed to provide teacher students with opportunities to learn from expert teachers, reflect on their own teaching behaviour and practise professional skills that would not otherwise be possible without this technology. Digital videos that are easy to use and various web platforms that facilitate the sharing and annotation of videos have opened up new opportunities for the development of novel learning environments in the field of teacher education (e.g. Sommerhoff et al., 2022). In the past few years, models for using videos recorded by mobile eye-tracking technology in teacher education have also been developed (e.g. Pouta et al., 2021).

Simulations are widely used in medical education, and a meta-analysis found that deliberate practise with simulations is superior to traditional clinical training in medical education (McGaghie et al., 2011). The use of simulations in teacher education is also gradually increasing. In their review of the use of simulations in teacher education, Theelen et al. (2019) synthesised the findings of 15 studies that applied computer-based simulations in teacher education. Several studies have demonstrated (Ferdig & Pytash, 2020; Samuelsson et al., 2022) that classroom simulations increase students’ self-efficacy and confidence in their teaching abilities. Classroom simulations were also found to have a positive impact on the development of classroom management skills.

New technologies can also be used in research on teaching and learning. Digitalisation has provided more ways to collect data and understand the teaching–learning process with multiple data channels and modalities. Multiple layers of data can be collected from contextual interactions, such as high-quality video data, psychophysiological measures and computer logs. With learning analytics, for example, these data can be used to create teacher dashboards, thus fulfilling students’ need for teacher scaffolds (Knoop-van Campen & Molenaar, 2020). Recently, eye-tracking technology has also been used to analyse teachers’ and student teachers’ abilities to notice relevant events in classrooms (Gegenfurtner et al., 2020; Pouta et al., 2021). Eye-movement technology, which has been used to model expert performance in other professional fields (Gegenfurtner et al., 2017), could also lead to promising training methods for teacher education.

## Articles in this special issue

Three of the articles (Basilotta‑Gómez‑Pablos et al., 2022; Peciuliauskiene et al.’s, 2022; Kulaksız & Toran, 2022) included in this special issue deal with digital competences and interventions aimed at enhancing them.

Basilotta‑Gómez‑Pablos et al. (in this issue) synthesised 56 studies on higher education teachers’ digital competences. The authors used special software called SciMAT to analyse the content of the articles and to present thematic networks. Their review of the literature revealed that the topic is timely and that the number of relevant studies is increasing rapidly. The reviewed studies generally relied on teachers’ self-reports and self-evaluations of their abilities. Overall, the results indicated that the participants were aware of their insufficient knowledge and skills in the area of digital technology. According to the synthesis, many of the articles describe teachers’ experiences of various projects and activities aimed at improving their digital competences; however, many of these articles describe informal learning using internet tools and social networks. The authors conclude that their review clearly shows the gap in the evaluation of teachers’ competence in teaching and learning practice. Their recommendation is that more interventions and training programmes be created to support the development of teachers’ digital competence.

- The recent challenges in education caused by the pandemic situation raised teachers’ awareness on the gap of their digital skills.
- Despite the developed national or EU digital competences frameworks the trend remains that the development of digital skills is not systemic and lacks coherence in in-service and pre-service teacher education.
- Further studies may bring more insights regarding more effective interventions to teaching practices with a wider application of digital technologies.

Peciuliauskiene et al.’s (2022) paper presents the results of their survey of two Lithuanian universities that offer teacher education programmes. Their questionnaire focused on information literacy (search and evaluation) and ICT self-efficacy. According to their results, both information literacy variables predicted teacher students’ ICT self-efficacy. Additionally, there was an indirect relationship between information evaluation and ICT self-efficacy. The findings of the study are discussed in terms of their theoretical and practical implications. The research indicates that information search ability does not depend on a person’s digital nativity, contrary to what is sometimes assumed when referring to the younger generation of pre-service teachers. As an ICT literacy component, information evaluation has become particularly pertinent during the COVID-19 situation and recent challenges related to distinguishing credible information from the vast amount of fake news and propaganda. It is also noted that optimal time and resources should be planned for the development of information search and evaluation abilities; however, more time should be allocated for the development of information search literacy, as it directly predicts pre-service teachers’ ICT self-efficacy. Based on the findings of this study, we identify the following trends and implications for further studies:

- ICT self-efficacy of teachers contribute to the enhancement of teaching and learning process however, ICT self-efficacy should not be limited to specific ICT skills but rather on rethinking the organisation of the teaching process and rethinking the principles of teaching. In other words, the development of digital skills alone without integrating them with specific pedagogical content knowledge and teaching strategies would be less beneficial.
- Further studies could be focused on how digital skills development could be better aligned with the development of teacher pedagogical strategies and specific subject areas.

The starting point of Kulaksız and Toran’s study (2022) was the observation that, despite pre-service teachers’ participation in courses on ICT integration, these teachers are still not confident about their competences to apply their knowledge in practice. In their study, Kulaksız and Toran used the so-called praxeological approach, which aims to produce beneficial knowledge and skills and to organise a democratic and participatory environment. The results indicate that the participants were prepared to transform their skills into practical pedagogical situations due to the personal development they experienced during and after the completion of the co-created course. As pre-service teachers could co-create the technology course, this allowed them to develop not only digital competences but also self-regulated learning skills, collaborative project development skills and peer mentoring skills, which contributed to building their sustainable motivation—an important component of teachers’ self-efficacy. This article highlights that.

- Teachers’ motivation is increased through participatory design in their professional development practices which allows to achieve a more holistic development of digital skills in combination with cognitive and non-cognitive competences.
- Further studies on how co-teaching contributes to digital skills development and innovative teaching strategies would allow to find more attractive models for teachers professional development.

The aforementioned three articles about digital competencies provide different perspectives on the issue, but they all emphasise the complexity of those competencies while also suggesting new approaches to deal with these challenges. In two of the articles, technology was not the focus of the studies but a method used in developing learning environments for teacher students.

In their study, Martin et al. (in this issue) investigated whether a video-based multimedia application about classroom teaching could be used to enhance teacher students’ professional vision. A teacher’s professional vision is their ability to observe and interpret important events in the classroom and determine the most appropriate teaching activities related to these events. Teacher education faces a variety of challenges because teacher students are unable to readily translate the knowledge they learn through formal teacher education into situation-specific skills that can be applied in actual classroom settings. The aim of the study was to help students make this translation with the aid of a video-based simulation developed using the findings of multimedia research. The simulation presented the classroom videos as short segments and provided prompts aimed at facilitating the students’ self-explanations. In the intervention study, they applied two versions of the video-based simulation: one with features based on multimedia research and one without these features. During the training, the segmented simulation with the self-explanation prompt resulted in increased noticing of relevant events in the teaching–learning process. In the comparison of pre-test and post-test results, all groups participating in the video training developed considerably in their professional vision, but the video simulation with the two multimedia elements did not differ significantly from the video training without these elements. The authors concluded that further research on the optimal implementation of the simulation is needed. Major issues raised by this article were:

- It confirms previous studies which have shown that the importance of the use of classroom videos in teacher education.
- When new methods (e.g. the multimedia elements added to the videos) are applied in interventions, it is important to pay attention to qualitative changes in learning processes and not only on immediate learning gains.
- The results also indicate that methods which have been effective in one context do not necessarily work in a new environment.

Nickl et al.’s study (in this issue) examined how video-based simulations can be used to enhance pre-service teachers’ assessment skills. The aim was to analyse individual learning processes in a simulated environment by taking into account learners’ cognitive and motivational-affective characteristics. Their study applied a person-oriented approach to analyse how these learner characteristics relate to students’ situated learning experiences and performance. In the latent profile analysis, three profiles were identified: one with high knowledge and average motivation-affect, one with high motivation-affect and average knowledge and one with below average knowledge and motivation-affect. Based on the results, it was confirmed that the motivated profile resulted in positive motivational experiences in the situation, while the knowledgeable profile resulted in relevant cognitive demands when working on the tasks. Situational experiences were also found to be related to learning outcomes when working with the simulation. In comparison to the other profiles, the cognitive profile demonstrated the most effective navigation and deep learning processes. The authors concluded that the identification of learner profiles is a promising approach that can uncover individual learner needs when working in technology-based learning environments. There lessons to learn from this study include:

- Learners prior learning and their personal characteristics can strongly mediate the outcomes of intervention programs
- Person oriented statistical analyses are promising approaches to focus on sub-groups with unique profiles.
- The challenge is to decide which individual characteristics are relevant in explaining varying effects of interventions.

## Practices which stimulate change

This collection of papers disclosing different aspects of the application of technology in todays’ teacher education clearly highlights that the development of digital competences should become an integral part of pre-service and in-service teacher education. In line with the holistic view on teacher competencies (Metsäpelto et al., 2022), this special issue suggests that digital competences appear as a strong component within cognitive and non-cognitive competences that contribute to high-quality teaching.

The results of the studies presented in this special issue strongly reflect recent studies (Falloon, 2020; Lin et al., 2022) demonstrating that the development of mere digital skills is not sufficient; we should instead structure teacher education to promote the development of digital teaching competences, including ICT attitudes, ICT skills, data literacy and deep pedagogical understanding of the opportunities and limitations of the use of technology in education. Digitally competent teachers are more capable of integrating technologies into their regular teaching practices while also creating more appropriate conditions for personalised learning (Schmid & Petko, 2019). This is important because large international evaluation studies (OECD, 2014, 2019, 2020) have shown that the inadequate use of technology can be harmful for student learning.

It should also be noted that the COVID-19 pandemic created additional challenges for teachers and contributed to changes in their teaching practices and digital habits (Blume, 2020). Reflecting school situations caused by COVID-19, numerous studies from the last 2 years have revealed a much wider scope of application of digital technologies in education and a need to create active interactions with learners (Greenhow et al., 2021). They have also identified a widening gap between learners who are more digitally advanced and less digitally competent teachers (Blume, 2020).

Likewise, simulations and other technological applications can be used to provide richer learning opportunities in teacher education. These new tools can help develop more effective models to connect theoretical content and practical skills. A big challenge for teacher education is to create opportunities for students to deliberately practise skills that are needed in classroom teaching while at the same time deepening student teachers’ theoretical understanding of teaching–learning processes.

## Data Availability

The paper is based on openly available data.
